# Reimagining community relationships for organizational learning: a scoping review with implications for a learning health system

**DOI:** 10.1186/s12913-021-06640-9

**Published:** 2021-06-27

**Authors:** Crystal Milligan, Whitney Berta

**Affiliations:** Institute of Health Policy, Management and Evaluation, University of Toronto, 155 College Street, Suite 425, Ontario M5T 3M6 Toronto, Canada

**Keywords:** Learning health system, Organizational learning, Learning organization, Community

## Abstract

**Background:**

Communities represent a highly relevant source of knowledge with regard to not only healthcare performance but also sociocultural context, yet their role in learning health systems has not been studied. Situating the learning health system as an organization, this paper explores the phenomenon of organizational learning from or with communities (defined as one of ‘the people,’ such as a town, a specific patient group or another group directly receiving a healthcare service).

**Methods:**

We conducted a scoping review to determine what is known about organizational learning from or with communities that the organization serves, and to contribute to a more comprehensive evidence base for building and operating learning health systems. In March 2019, we systematically searched six academic databases and grey literature, applying no date limits, for English language materials that described organizational learning in relation to knowledge transfer between an organization and a community. Numerous variables were charted in Excel and synthesized using frequencies and thematic analysis. We updated this search in August 2020.

**Results:**

In total, 42 documents were included in our analysis. We found a disproportionate emphasis on learning explicit knowledge from community rather than on tacit knowledge or learning in equal partnership with community. Our review also revealed inconsistently defined concepts, tenuously linked with their theoretical and empirical foundations. Our findings provide insight to understand the organization-community learning relationship, including motives and power differentials; types of knowledge to be learned; structures and processes for learning; and transformative learning outcomes.

**Conclusions:**

Our review makes a singular contribution to organizational learning literatures by drawing from diverse research disciplines such as health services, business and education to map what is known about learning from or with community. Broadly speaking, learning health systems literature would benefit from additional research and theory-building within a sociological paradigm so as to establish key concepts and associations to understand the nature of learning with community, as well as the practices that make it happen.

**Supplementary Information:**

The online version contains supplementary material available at 10.1186/s12913-021-06640-9.

## Background

There is much yet to learn about the learning health system, a variously defined concept whose hallmark characteristics are poorly articulated. Taking a complex adaptive systems view, we conceptualize the learning health system as an organization whose learning is generated by internal and external interactions and relationships [1, 2]. In organizational learning theory, the idea that organizations learn from knowledges in their internal as well as external environments is well established [3]. Theories of social learning further characterize knowledge and learning as context- and relationship-dependent [4–7].

Yet surprisingly, when it comes to studying learning phenomena in healthcare, the organization’s relationships with communities it serves are overlooked. In fact, community relationships represent the least researched dimension of relationship-centred healthcare [8]. In this light, we conducted a scoping review to determine what is known in extant literatures about organizational learning from or with what is arguably the most important stakeholder in healthcare: the community, which we define as one of ‘the people’—such as a town or a specific healthcare patient group.

Though the rhetoric of community engagement may be strong in healthcare, there is a tendency to refer to organizational learning from these interactions in the abstract [9]. The result is an obscured understanding of the motives, structures, processes and outcomes of learning by a learning health system. This leaves open the possibility to confuse ‘learning from’ and ‘learning with’ community, where ‘learning from’ community entails the organization extracting data about the community and ‘learning with’ community entails authentic partnership, power-sharing and the co-production of knowledge.

Scoping reviews are well-suited to studies that aim to map concepts across interdisciplinary boundaries and review the range of evidence [10]. Following established methodology [11–13], our review contributes to an expanded view of organizational learning and a more comprehensive evidence base for building and operating learning health systems, drawing from health services research and other research disciplines including the organization sciences, business, and education. In this way, we shed light on relationships beyond the learning health system’s organizational borders, namely with the communities the health system serves. Our findings underscore the importance of meaningful community involvement pursuant to the adage, “nothing about us, without us.”

## Methods

### Search strategy

Our search included an academic database search, hand searching of relevant journals, search engine queries, targeted website review and reference tracking. We based our search on three concepts: organizational learning, including social learning; the learning organization, including learning health systems; and community. Additional File 1 contains theoretical background on these concepts. Moreover, the search protocol is registered and available with the Open Science Framework (https://osf.io/uv9b8).

The initial academic search strategy was developed for Ovid MEDLINE® (see Additional File 2) and then translated for other databases. Several preliminary, iterative searches permitted us to refine a search using a combination of subject headings and text words, and tailor to each database. Final searches with no language or date limits were conducted on March 19, 2019 in six databases. The lead author also hand searched all issues of two journals.

We searched online, using numerous combinations of key terms in Advanced Google Search and Duck Duck Go. The lead author reviewed search results up to five webpages after the last hit. Websites of four organizations known for their contributions to studying learning health systems were also browsed and searched. When multiple publications were available as part of a series, the most recent or summative publication was chosen. In the case of one evidence synthesis publication found in this way, the lead author found ten additional potentially eligible citations through reference tracking. Table 1 lists our information sources. All potentially eligible citations were loaded into EndNote X9.1.1 for deduplication [14].

**Table 1:** Summary of search locations

**Academic databases**	**Online search engines**
• Ovid MEDLINE® • PsycINFO • CINAHL Plus • ERIC • Web of Science • Business Source Premier	• Advanced Google Search • Duck Duck Go
	**Targeted websites**
	• Agency for Healthcare Research and Quality (ahrq.gov) • McMaster Health Forum (mcmasterforum.org) • National Academy of Medicine (nam.edu) • The Learning Healthcare Project (learninghealthcareproject.org)
**Hand searched journals**	
• *The Learning Organization* • *Learning Health Systems*	
**Reference tracking**	
• Reviewed reference list of 1 evidence synthesis report	

### Eligibility criteria

Citations had to fulfill the following criteria to be included in this study:

- Description of interaction or knowledge transfer between an organization and a community;
- Reference to organizational learning, a learning organization or learning health system;
- Description of the relationship between the organization and community where the community should benefit from organizational outputs.

Provided that they met these criteria, citations were included based on an inclusive understanding of ‘community’ (e.g., client, customer, patient, consumer, public). Citations were excluded if they: focused on interorganizational learning or professional communities of practice; described learning at a level other than the organization; were not in English; or offered insufficient evidence, typically because they were book reviews or short commentaries.

### Study selection

**Fig. 1:**
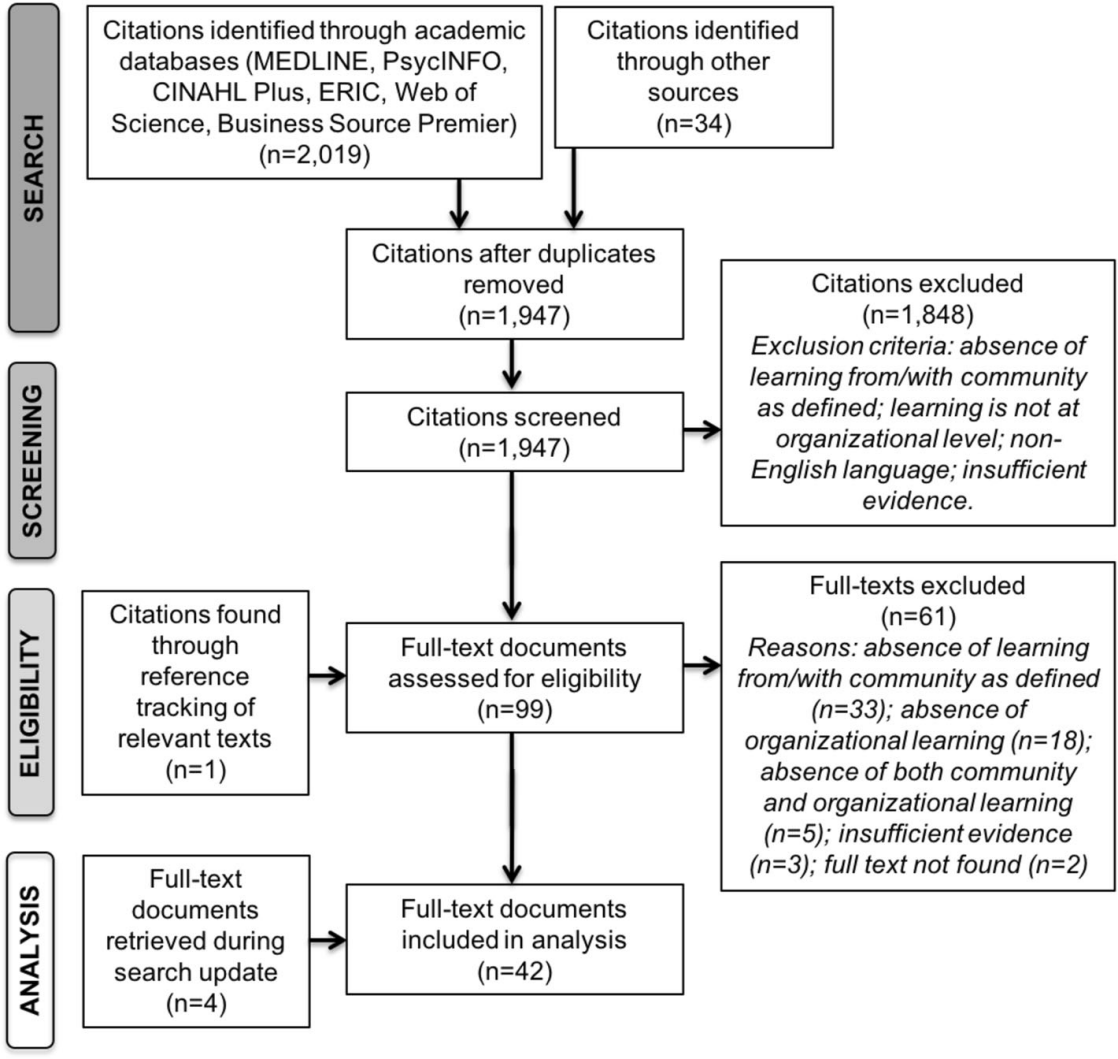
Flow chart of study selection

The lead author and a second reviewer conducted a first phase of screening using a web-based tool [15]. Over four rounds, the two reviewers screened the titles and abstracts of 524 (27 %) citations with the objective to reach consistent inter-rater agreement above 80 %. Agreement in each successive round was 74 %, 81 %, 83 % and 92 %, respectively, with conflicts resolved through discussion. The lead author screened the remaining 1,423 (73 %) titles and abstracts independently. Where there was insufficient or unclear information to exclude a citation, the citation was included for full-text review. In total, 98 full texts were reviewed by the two reviewers. Disagreements were resolved by consensus. Another citation was included at this stage after reviewing the reference lists of all documents. Our process is illustrated in Fig. 1.

### Data charting and collation

Using an Excel chart that had been pilot tested on five included texts, the lead author extracted data for numerous variables including article characteristics (e.g., discipline, study design, objective, findings); theoretical frameworks and definitions; dimensions of learning (e.g., processes, structures, motives, outcomes); community variables (e.g., conceptualization of community and community roles); and power relations. Results were synthesized using frequencies and thematic analysis [16]. Meta-analysis was not performed.

### Search update

We updated our results in August 2020. The lead author performed the same search detailed above, limiting results to items published in 2019 or 2020. The lead author performed study selection and data charting independently, applying the same eligibility criteria as above.

## Results

We assessed 1,947 titles and abstracts and 99 full-text documents for eligibility during the initial search in March 2019. Of these, 38 full-text documents fulfilled our criteria. During the study update of August 2020, four texts were added [17–20]. We therefore analyzed 42 full-text documents from the research disciplines of health services, business, natural resource management, organization and management sciences, education and social services (Table 2). For more detail, see Additional File 3.

**Table 2:** Document characteristics (*n* = 42)

Publication year	1995-1999	3	(7.1%)
	2000-2004	7	(16.7%)
	2005-2009	4	(9.5%)
	2010-2014	11	(26.2%)
	2015-2018	17	(40.5%)
Research discipline	Business	15	(35.7%)
	Health services	13	(31.0%)
	Natural resource management	5	(11.9%)
	Organizations and management science	5	(11.9%)
	Education	2	(4.8%)
	Social services	2	(4.8%)
Focus	Organizational learning	24	(57.1%)
	Social learning	3	(7.1%)
	Learning organization	7	(16.7%)
	Learning health system	8	(19.0%)

### Detachment from theory

Not one healthcare text drew from theory in any research discipline in defining either ‘organizational learning’, ‘social learning’, ‘learning organization’ or ‘learning health system’. The learning health system texts were strikingly detached from theory, instead citing practice-based definitions and frameworks encompassed in National Academy of Medicine reports (e.g., [21–23]).

### Learning from, and learning with, community

**Fig. 2:**
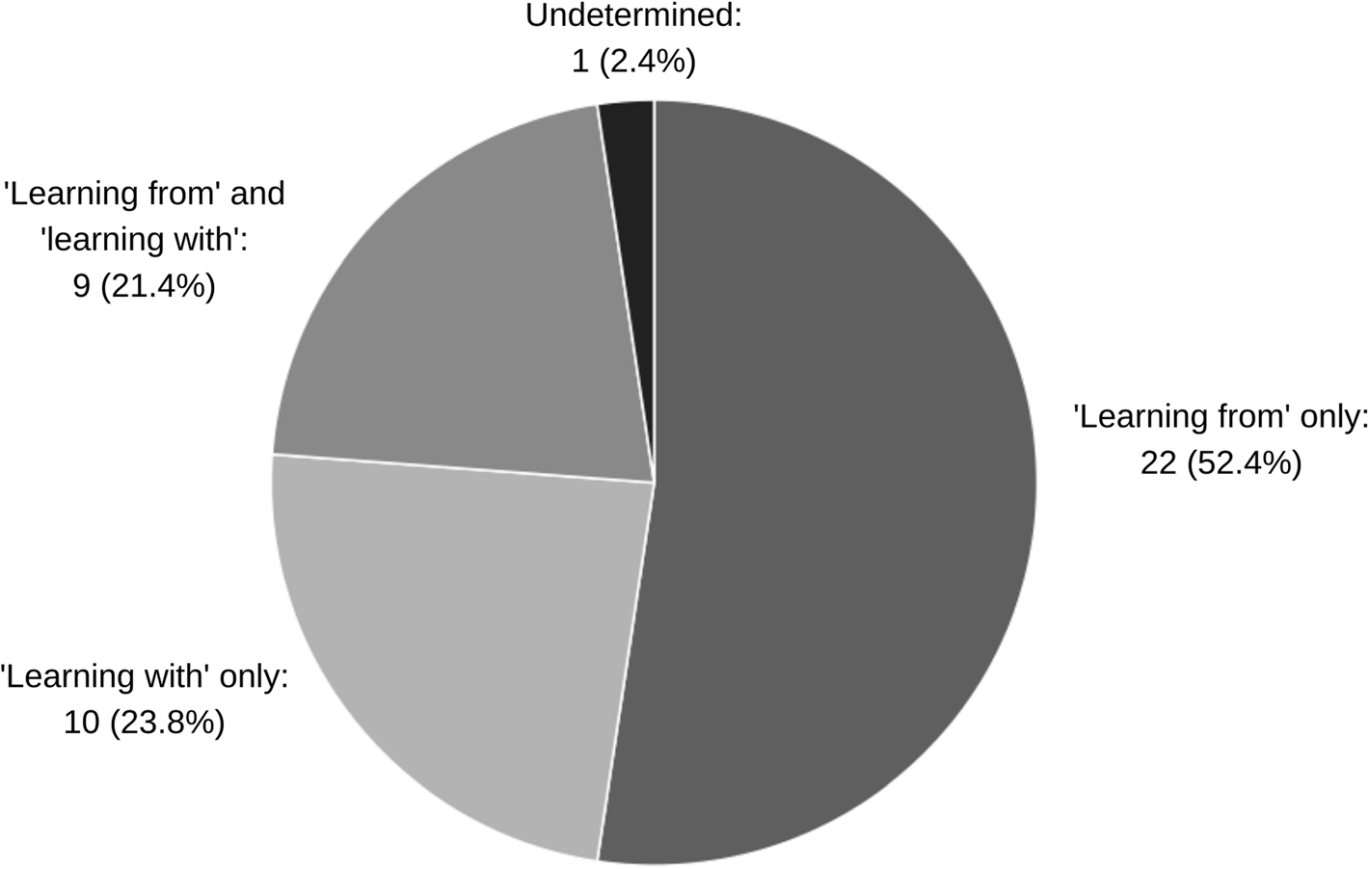
Focus on ‘learning from’ community, ‘learning with’ community or both, by number of citing texts

As in Fig. 2 and 10 (24 %) texts focused on learning with community [23–32]. Some authors associated the quality of organizational learning with the quality of dialogue and engagement with the community [30, 33, 34]. They implied that although learning with community requires more effortful participatory methods [33], they are preferable to gathering information about the community without its involvement.

In comparison, more than half the documents (*n* = 22, 52 %) described learning from community without community partnership, typically through the collection and management of information about the community. Healthcare texts tended to focus on learning through patient data collection. Business texts tended to discuss collecting data about customers to learn their needs, preferences and spending patterns.

Nine (21 %) texts suggested that learning from and learning with community was possible concurrently [18, 33–40].

### Knowledge transfer

Thirty-four (81 %) texts explored the nature of knowledge transfer between the community and organization. Among these, four healthcare texts [41–44] and three business texts [20, 45, 46] emphasized administrative or research data as drivers of organizational learning. Alternatively, 17 texts suggested that direct interaction and relationships—such as volunteering or board membership [25, 47] or participation on advisory councils [26, 27]—drove learning from community knowledges. The authors of six documents spoke about open, cross-boundary dialogue to facilitate the flow of ideas [29, 32, 40, 48–50]. Similarly, three articles suggested that there must be an open space of discourse for community evidence to mix with organizational knowledge and help to transform organizational frames of reference [31, 38, 40].

Five texts incorporated the concepts of tacit and explicit knowledge [19, 29, 32, 49, 51]. One suggested that knowledge sharing is easier between different groups when they already share common tacit knowledge [51] and another described how tacit knowledge is externalized through dialogue [19].

### Learning motives

**Fig. 3:**
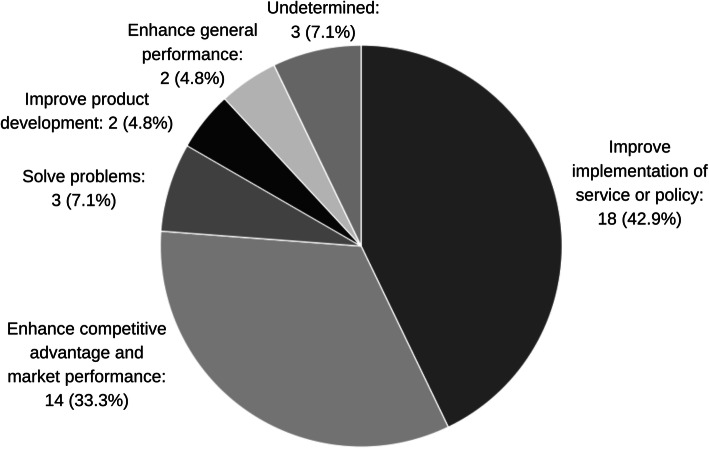
Stated motives for learning from or with community, by number of texts

The documents revealed a wide range of motives for organizational learning from or with community. These motives were sometimes implied in the definitions authors used to describe learning. For instance, unlike definitions for ‘learning organization’, ‘organizational learning’ or ‘social learning’, definitions for ‘learning health system’ were the only ones not to consider transforming underlying organizational norms, assumptions or behaviour [26, 27, 33]. Other times, the motives for learning from or with community were clearly articulated (Fig. 3). Eighteen (43 %) documents, including all those focused on learning health systems, cited desire to improve the implementation of a service or policy as the purpose of learning. Fourteen (33 %) documents, nearly all from business research, cited desire to enhance competitive advantage and market performance. By contrast, three of five natural resource management articles cited desire to solve societal problems [38, 39, 52].

Authors seemingly took for granted that organizations would achieve outcomes (e.g., improved service) that matched their motives (e.g., to improve service). We found insufficient detail regarding the processes linking motives to outcomes.

### Structures and processes

All texts except two [49, 53] contemplated structures to support organizational learning from or with community. Kass and Faden [42] implied that in healthcare, the structure for organizational learning is, in fact, the learning health system, yet did not describe this structure beyond its obligations and guiding principles. For their part, Reid and Hickman [34] argued that organizational learning depends on participatory structures and relationships. Nonaka et al. [32] described a common space or context shared between individuals and groups where knowledge is jointly created, shared and utilized; this shared context, they suggested, can be a physical or virtual space with fluid boundaries.

Across all documents, the distinction between structures and processes for learning was unclear. For the most part, authors described structures like patient advisories or boards in general terms; they seemingly assumed that beneficial learning processes would follow but did not describe them. We noted that definitions given for ‘learning health system’ prioritized learning through research. Furthermore, informatics and digital data infrastructure were part of the definitions offered by three documents only, each of which pertained to learning health systems [23, 26, 41].

### Strategies

**Fig. 4:**
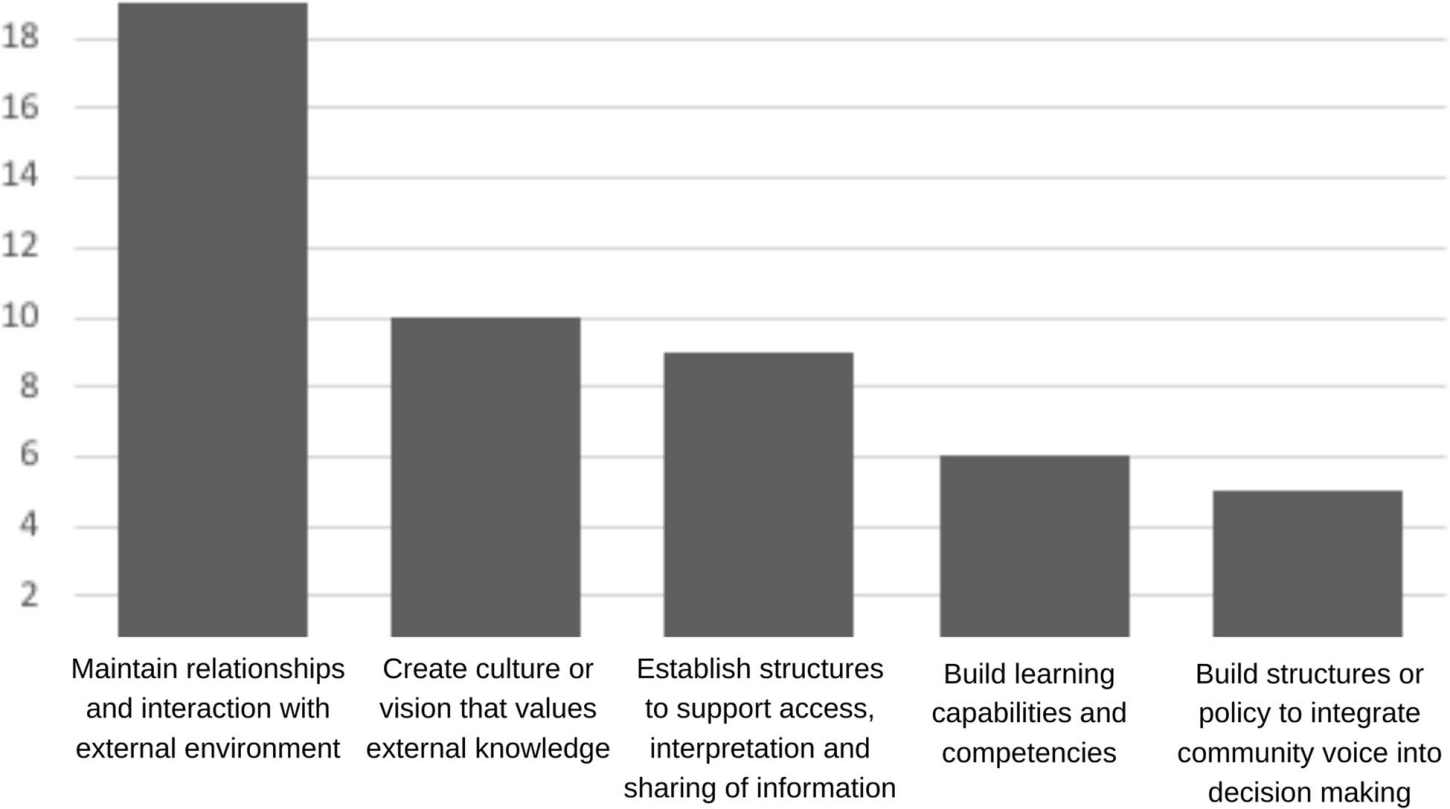
Strategies to support organizational learning from or with community, by number of citing texts

The maintenance of relationships and social interaction was the most frequently cited strategy to support organizational learning from or with community, clearly mentioned in 19 (45 %) texts (e.g., [24, 25, 32, 37, 48, 54]). Ten (24 %) texts recommended creating a culture or vision that openly values external knowledge (e.g., [23, 36, 43, 55]). Nine (21 %) texts recommended designing internal structures to support access, interpretation and sharing of information (e.g., [19, 27, 39, 43, 45]). These strategies are depicted in Fig. 4.

### Power relations

Among the 10 (26 %) texts that explored power relations between the organization and community [24, 25, 30, 31, 36–40, 56], trust and transparency were prominent themes. Equal partnership was highlighted as a foundation for community trust toward large organizations [30, 36]. This sentiment was repeated by others who felt that balanced power relations were necessary for organizational learning outcomes benefitting the community. Whereas trust should be earned, they said, organizations typically enter into community relationships holding significant unearned control and privilege [37].

### Distinct knowledge systems

Four (10 %) texts paid considerable attention to the position of community knowledge systems relative to organizational, professional or scientific knowledge [31, 38–40]. They suggested that local knowledges are habitually devalued compared with explicit, university-based scientific knowledges, thus restricting the ability of communities to participate in learning processes.

### Ethics

Four (10 %) healthcare texts raised ethical concerns that emerged from a focus on research, specifically the tension between patient privacy and disseminating research results [26, 36, 41–43]. Psek et al. [26] questioned whether patient care and research could operate under joint ethical frameworks. Other ethical debates considered which organizational learning activities require ethical approval [43], how patients engage in research [42, 43], and how to translate research results so they benefit patients [41, 42].

## Discussion

### Value in community

If the goal is to improve the health of a population—not simply perform within the confines of organizational boundaries and compete with other healthcare organizations—it seems logical to consider learning at multiple system levels, including with community. This should give health services researchers and practitioners pause to reflect on their approaches. What a learning health system learns, how it learns, and to what effect are intimately tied with who contributes to learning. Diversity, not merely in membership but also in discourse, cultivates ideas, drives adaptation and leads to justified outcomes [57, 58].

Yet the value of learning with community extends beyond simply diversifying the knowledge base of organizations. Communities extend our conceptualization of what counts as evidence. Striving toward evidence-based medicine and decision making, our reliance on purely explicit knowledge, stripped from context in the name of objectivity, provides only a partial picture. Communities represent knowledge that is discrete from organizational, scientific or other forms, providing insight into healthcare performance at organizational and whole system levels; surely, their knowledge counts as evidence, too.

### Reimagining organization-community relationship

Direct relationships and knowledge-intensive interactions between people are needed for organizations to overcome barriers to learning from local knowledge [59], but it is not apparent what the organization-community learning relationship might look like. We noted throughout this review that ‘relationship’ means many things in the literature, from connection and interactions between different parties, to a one-way flow of data about one party to the other. With diverse meanings and models of relationship in play, it is unclear which kinds of relationships best support organizational learning. Furthermore, there is little guidance regarding how to establish learning relationships with communities.

We discovered a tendency to concentrate on collecting information about community (e.g., [44–46, 48])—or ‘learning from’ community—which risks reducing the community’s position to that of merely ‘being known’ rather than enhancing community capacity to ‘know.’ It follows that ‘learning with’ community is a defining characteristic of the relationship in which organization and community alike are empowered to exchange their knowledges and ways of knowing, on equal footing. This line of thinking offers conceptual clarity for building and nurturing organizational learning relationships with communities. What remains is to apply these concepts to theoretical and empirical analyses of organizational learning and learning health systems.

### Ways of learning

The learning health systems texts included in this review suggested a bias toward quantified, explicit and research-derived knowledge. Such a narrow orientation toward clinical research and health service data neither adequately captures the breadth and depth of relevant theory from other research traditions, nor promotes a model that gives credence to non-clinical or tacit forms of knowledge. The focus on formal research raises a number of ethical questions, including those regarding whose purposes are served (do patients define research questions?); structures of learning (will research be integrated with organizational practice, or siloed?); and sources of learning (do organizations learn from patient registries or patient encounters?). Arguably just as problematic, recent study suggests that a minority of community members feel any responsibility to participate in healthcare research [60].

We speculate that the overwhelming propensity for research and evidence-based medicine could inadvertently, and perversely, steer health system planning away from models of relationship-centred care and toward a penchant for explicit research-based knowledge over tacit knowledge from other sources. Healthcare systems already field criticism for performance frameworks that overemphasize explicit measures, perceived as forsaking service for targets [61, 62]. The learning health system may be best supported by a combined approach of emergent organizational learning and deliberate research, with structures in place to support both processes.

### Knowledge and power

The extent to which an organization can learn with communities is influenced by an in-built power imbalance [63]. Studies of healthcare organizations have found that co-opting power through the manipulation of supposedly participatory mechanisms erodes community trust and hinders the co-production of knowledge [64–66]. In other words, if a partnership is founded in rhetoric more than reality, the extent to which the organization can listen and learn from communities is unclear. Learning with community would require an organization to be comfortable with the possibility that new evidence leads to the transformation of underlying assumptions and values, and the sharing of knowledge and power.

### Learning as transformation

Whereas transformation may not always be preferable or lead to the best outcomes for an organization [67], there are nonetheless real reasons to contemplate transformation of the structures, processes, values and assumptions that underlie healthcare organizations, such as the need to improve the accessibility and quality of care for systemically marginalized groups. There is an opportunity for further study and reflection to gauge the value of such transformation through learning, and how this might be supported as a relationship-centred, collaborative process.

### Looking inward to learn with others

Looking inward is fundamental for the organization, not only as part of a self-reflexive practice, but also to establish internal structures and processes for the interpretation, dissemination and integration of external knowledge. A single-minded mentality of knowledge acquisition, via data extraction about or from the community, may preclude relationship with that community. Managers must therefore design meaningful interactions that facilitate sharing of explicit and tacit knowledge, within and outside the organization. They must also create favourable conditions for learning that include judgement-free spaces in which to share ideas, as well as a vision that values learning in general and learning with community specifically. To respect and maintain the integrity of community knowledges, the organization would need to partner with the community to set up initial structures, processes and boundaries for collaborative learning and then be open to changes the community may suggest over time.

### Opportunity for theory-building

Learning health systems literature is essentially atheoretical. Additional investigation to explore and verify associations between social relationships, structures, processes, types of knowledge and outcomes would contribute to defining characteristics of a learning health system, and possibly clarify which types of learning (e.g., learning from or with community), driven by which motives, lead to better outcomes.

### Strengths and limitations

We endeavoured to make a singular contribution to learning health systems and other organizational learning literatures by drawing from diverse research disciplines. Consistent with scoping review methodology [10, 11], we did not critically appraise the reviewed texts. We therefore avoided making inferences in our results beyond what was clearly stated by the authors. Furthermore, given the current dearth of literature, we did not differentiate between community as a collective and community as represented by an individual. This distinction could be made in future research. Finally, this article does not incorporate consultation with community partners, which would be invaluable.

## Conclusions

There is substantial work still left to do if we are to progress the concept of a learning health system from the abstract into practice. With this in mind, we looked across research disciplines and shone a spotlight on the communities that an organization serves. Our results encourage a new way of thinking about the learning health system: not solely as an organizational entity, but perhaps as a network of relationships. Future research can explore whether the learning health system may be, by definition, one whose culture, structures and processes afford a meaningful shared context for social interaction and knowledge co-creation with the communities it serves, with borders extending beyond the traditionally defined organization. Such would be a context wherein the organization’s relationships are defined by true partnership in place of rhetoric.

## Supplementary Information

**Additional file 1.** Theoretical Background of Key Concepts.

**Additional file 2.** Sample Search Strategy

**Additional file 3.** Data Charting

## Data Availability

The datasets used and/or analysed during the current study are available from the corresponding author on reasonable request.
