# Supplementary material for: Reimagining community relationships for organizational learning: a scoping review with implications for a learning health system
Source: BMC Health Serv Res. 2021 Jun 27;21:603. doi: 10.1186/s12913-021-06640-9 (PMC8237504; doi:10.1186/s12913-021-06640-9)
Supplement: Supplementary file 1 — Additional file 1. Theoretical Background of Key Concepts. [file 12913_2021_6640_MOESM1_ESM.pdf]

Additional File 1: Theoretical Background of Key Concepts

As a starting point, we conceptualized learning health systems as organizations that are complex adaptive systems, intrinsically connected with their internal and external environments. In this view, system behaviour and learning are generated by complex interactions and relationships among the system's constituent parts and internal or external agents [1, 2]. Critical to enabling these interactions and relationships is what complexity science theorists call adaptive space, which is described as temporary network structures that open channels of communication such that diverse ideas can mix and learning can emerge [3]. It follows that community knowledge from the external environment can contribute to organizational learning provided the structures and relations of power within adaptive space permit that knowledge to be valued and integrated.

Argyris and Schön [4, 5] described organizational learning as a process of error correction via single-loop or double-loop learning, where double-loop learning goes beyond simply solving a problem to changing underlying assumptions or values that may alter future behaviour. They proposed that individual learning embeds within organizational memory and thus can lead to organizational changes and performance improvement. Similarly, Huber [6] suggested that organizational learning comprises knowledge acquisition, information distribution, information interpretation and organizational memory, frequently resulting in a change in the nature and range of the organization's potential behaviours.

Tension between individual and collective initiatives, including learning, is a regular feature of organizational learning literatures across research disciplines. The rise of social theories of learning in the 1990s led to a more collective-oriented ontology of organizational learning that was less dependent on individual learning as its source [e.g., 7, 8]. The natural resource management sciences, in particular, embrace the concept of social learning. Drawing from social theories that characterize learning as active social interaction and participation [8, 9], social learning is defined as “a change in understanding that goes beyond the individual to become situated within wider social units” [10]. In our interpretation, organizations count as ‘wider social units’ given that they are social entities—albeit coordinated in a hierarchical fashion. Yet again, the ‘change in understanding’ is frequently clarified with transformative learning theory [11], a body of theory initially focused on individual learning but regularly applied to explain the transformation of assumptions and worldviews that comes with second-order, or double-loop, learning [10, 12].

We view the learning health system as a kind of learning organization. The learning organization concept originates in work by Argyris and Schön [4, 5] but was popularized by Senge [13] in North America and Burgoyne, Pedler and Boydell [14] in the United Kingdom. Senge [13] defined the learning organization as one with both adaptive (single-loop) and generative (double-loop) learning capacities built on individual, collective and organizational learning processes. Notably, the learning organization is not equivalent to organizational learning. Many authors describe organizational learning and the learning organization as the process and product, respectively—a normative distinction between what is done and what should be [15-17].

Others have suggested that the two concepts are developing along divergent tracks, with learning organization theorists pulling concepts from organizational learning, but not the other way around [18]. Meanwhile, scholars highlight a lack of consensus in the way organizational learning and learning organizations are described, with authors often confusing process with outcome [19] or failing to recognize how different worldviews may pre-determine how organizational learning is conceptualized within different research disciplines [20].

In this review, we subscribe to a conceptualization of the learning organization that combines three elements: organizational knowledge, including internal and external knowledge; knowledge management, or the control of structures and processes to facilitate knowledge sharing; and organizational learning processes [15, 21].

For those readers who wish to delve further, there are many excellent reviews of the origins and evolution of organizational learning scholarship [e.g., 18, 20, 22-24].

References

1. Lichtenstein BB, Uhl-Bien M, Marion R, Seers A, Orton JD, Schreiber C: **Complexity leadership theory: An interactive perspective on leading in complex adaptive systems**. *Emergence: Complexity & Organization* 2006, **8**(4):2-12.
2. Plsek PE, Greenhalgh T: **Complexity science: The challenge of complexity in health care**. *BMJ: British Medical Journal* 2001, **323**(7313):625-628.
3. Uhl-Bien M, Arena M: **Complexity leadership: Enabling people and organizations for adaptability**. *Organizational Dynamics* 2017, **46**:9-20.
4. Argyris C, Schön D: **Organizational Learning: A Theory of Action Perspective**. Menlo Park, CA: Addison-Wesley; 1978.
5. Argyris C, Schön D: **Organizational Learning II: Theory, method and practice**. Reading, MA: Addison-Wesley; 1996.
6. Huber GP: **Organizational learning: The contributing processes and the literatures**. *Organization Science* 1991, **2**(1):88-115.

7. Cook SD, Yanow D: **Culture and Organizational Learning**. *Journal of Management Inquiry* 1993, **2**(4):373-390.
8. Lave J, Wenger E: **Situated learning: Legitimate Peripheral Participation**. Cambridge: Cambridge University Press; 1991.
9. Wenger E: **Communities of Practice: Learning, Meaning, and Identity**. New York, NY: Cambridge University Press; 1999.
10. Reed M, Evely AC, Cundill G, Fazey IRA, Glass J, Laing A, Newig J, Parrish B, Prell C, Raymond C: **What is Social Learning?** *Ecology and Society* 2010:r1-r10.
11. Mezirow J: **Transformative Dimensions of Adult Learning**. San Francisco, CA: Jossey-Bass; 1991.
12. Yorks L, Marsick VJ: **Organizational Learning and Transformation**. *Learning as Transformation* 2000:253-281.
13. Senge PM: **The Fifth Discpline: The Art & Practice of the Learning Organization**. New York, NT: Doubleday/Currency; 1990.
14. Burgoyne J, Pedler M, Boydell T: **The Learning Company: A Strategy for Sustainable Development**. London, UK: McGraw-Hill; 1991.
15. Thomas K, Allen S: **The Learning Organisation: A Meta-analysis of Themes in Literature**. *The Learning Organization* 2006, **13**(2):123-139.
16. Örtenblad A: **On differences between organizational learning and learning organization**. *The Learning Organization* 2001, **8**(3):125-133.
17. Yeo RK: **Revisiting the roots of learning organization: A synthesis of the learning organization literature**. *The Learning Organization* 2005, **12**(4):368-382.
18. Easterby-Smith M, Araujo L: **Organizational Learning: Current Debates and Opportunities**. In: *Organizational Learning and the Learning Organization: Developments in theory and practice.* edn. Edited by Easterby-Smith M, Araujo L, Burgoyne J. London, UK: Sage; 1999: 1-22.
19. Örtenblad A: **What does "learning organization" mean?** *The Learning Organization* 2018, **25**(3):150-158.
20. Easterby-Smith M: **Disciplines of Organizational Learning: Contributions and Critiques**. *Human Relations* 1997, **50**(9):1085-1113.
21. Easterby-Smith M, Lyles MA: **Handbook of Organizational Learning and Knowledge Management**. Oxford, UK: John Wiley & Sons; 2011.
22. Prange C: **Organizational Learning - Desperately Seeking Theory?** In: *Organizational Learning and the Learning Organization: Developments in theory and practice.* edn. Edited by Easterby-Smith M, Araujo L, Burgoyne J. London, UK: Sage; 1999: 24-43.
23. Huysman M: **Balancing Biases: A Critical Review of the LIterature on Organizational Learning**. In: *Organizational Learning and the Learning Organization: Developments in theory and practice.* edn. Edited by Easterby-Smith M, Araujo L, Burgoyne J. London, UK: Sage; 1999: 59-74.
24. Tosey P, Visser M, Saunders MN: **The origins and conceptualizations of 'triple-loop' learning: A critical review**. *Management Learning* 2012, **43**(3):291-307.
