# Supplementary material for: Reimagining community relationships for organizational learning: a scoping review with implications for a learning health system
Source: BMC Health Serv Res. 2021 Jun 27;21:603. doi: 10.1186/s12913-021-06640-9 (PMC8237504; doi:10.1186/s12913-021-06640-9)
Supplement: Supplementary file 2 — Additional file 2. Sample Search Strategy [file 12913_2021_6640_MOESM2_ESM.pdf]

Additional File 2: Sample Search Strategy

Database(s): Ovid MEDLINE®: Epub Ahead of Print, In-Process & Other Non-Indexed Citations, Ovid MEDLINE® Daily and Ovid MEDLINE® 1946-Present

Search Strategy:

#	Searches
1	exp Learning/ and exp Organizations/
2	exp Social Learning/
3	(organi#ation and ("transformati* learning" or "double#loop learning" or "triple#loop learning" or "social learning" or "adaptive learning" or "high#order learning" or "high#level learning" or "meta#learning")).tw,kf.
4	("organizational learning" or "organisational learning").tw,kf.
5	1 or 2 or 3 or 4
6	(organi#ation* adj2 (knowledge or evidence or know-how)).tw,kf.
7	exp Knowledge Management/
8	(learning organization or learning organisation or learning health system).tw,kf.
9	5 or 6 or 7 or 8
10	(community involvement or client involvement or public involvement or patient involvement).tw,kf.
11	(community participa* or client participa* or public participa* or patient participa*).tw,kf.
12	exp Community-Institutional Relations/
13	(community collaborat* or client collaborat* or public collaborat* or patient collaborat*).tw,kf.
14	(community engag* or client engag* or public engag* or patient engag*).tw,kf.
15	(community consult* or client consult* or public consult* or patient consult*).tw,kf.
16	exp Community Networks/ or exp Community Participation/
17	(community partner* or client partner* or public partner* or patient partner*).tw,kf.
18	("customer knowledge" or "consumer knowledge" or "community knowledge" or "user knowledge" or "client knowledge" or "public knowledge" or "local knowledge" or "Elder knowledge" or "Indigenous knowledge" or "traditional knowledge" or "people* knowledge").tw,kf.
19	10 or 11 or 12 or 13 or 14 or 15 or 16 or 17 or 18
20	9 and 19
