# Supplementary material for: Reimagining community relationships for organizational learning: a scoping review with implications for a learning health system
Source: BMC Health Serv Res. 2021 Jun 27;21:603. doi: 10.1186/s12913-021-06640-9 (PMC8237504; doi:10.1186/s12913-021-06640-9)
Supplement: Supplementary file 3 — Additional file 3. Data Charting [file 12913_2021_6640_MOESM3_ESM.pdf]

Additional File 3: Key characteristics of learning described by 42 full-text documents included in review

Citation	Motive to learn	With/from community	Community	What is learned	Learning outcomes	Structure for learning	Practice or process of learning	Role of organization	Role of community
Learning health system documents									
Kass & Faden (2018)	Improve implementation of service or policy	From	Service or product user	Patient treatment and outcomes	Better care	The learning health system	Collection, aggregation, analysis and translation of patient data	Collect information & manage	Allow use of information
Key & Lewis (2017)	Improve implementation of service or policy	With and from	Service or product user	Community perspectives	Continuous learning	Communication and collaborative engagement	Continuous quality improvement	Increase participatory engagement; take time for trust building	Ask (for) more
Lavis et al (2018)	Improve implementation of service or policy	From	Service or product user; all communities (geographic, cultural, etc)	Information from the patient, clinical practice or policy	Continuous learning	Communication and engagement; information technology	Patient-centered focus	Increase participatory engagement; instill learning culture; generate evidence	Allow use of information
Lowes et al (2016)	Improve implementation of service or policy	From	Service or product user	Patient outcomes and research data	Reduced healthcare utilization and reduced costs	Information technology	Translational research	Collect and manage information	Allow use of information
McGinnis et al (2013)	Improve implementation of service or policy	With	Service or product user; public; all communities (geographic, cultural, etc)	Knowledge from every patient interaction, treatment and service	New clinical knowledge and better care	Communication and collaborative engagement	Continuous feedback	Increase participatory engagement; promote learning structures and culture	Co-design, co-production

Citation	Motive to learn	With/from community	Community	What is learned	Learning outcomes	Structure for learning	Practice or process of learning	Role of organization	Role of community
Murphy & Patlak (2010)	Improve implementation of service or policy	From	Service or product user	Information about patient treatments and outcomes	New knowledge and evidence embedded into standardized care	Information technology	Generation of scientific evidence and embedding in practice	Collection and manage information; exploitation	Allow use of information
Psek et al (2015)	Improve implementation of service or policy	With	Service or product user	Data and patient experience	Improved service	Communication and collaborative engagement	Engagement and partnership	Maintain relationships with community	Provide input and feedback
Teare et al (2018)	Improve implementation of service or policy	With	Service or product user	Patient and family perspectives	Integration of research and quality improvement	Communication and collaborative engagement	Collaboration rather than consultation	Increase participatory engagement	Not described
Learning organization documents									
Fojt (1995)	Enhance competitive advantage and market performance	Unclear	Service or product user	Information about customers and competitors	Sustainable competitive advantage	Communication and collaborative engagement	Communication and cooperation	Instill learning culture	Not described
Jarvis et al (1997)	Improve implementation of service or policy	With	Service or product user	Information to improve the service	Improved service	Communication and collaborative engagement	The Five Disciplines (Senge 1990	Increase participatory engagement	Participate
Kelsey (2003)	Improve implementation of service or policy	With	Stakeholder/ interested party	Knowledge of all types	Problem-solving	Communication and collaborative engagement	Ongoing connection and reflection	Recognize community value	Participate Share knowledge

Citation	Motive to learn	With/from community	Community	What is learned	Learning outcomes	Structure for learning	Practice or process of learning	Role of organization	Role of community
Ochocka et al (1999)	Improve implementation of service or policy	With and from	Service or product user	Information about the service user as a whole person	Changed foundational values	Communication and collaborative engagement	Participatory processes and relationship-building	Increase participatory engagement; be open to change	Participate in advisories
Reid & Hickman (2002)	Improve implementation of service or policy	With and from	Service or product user	Simple data or information that changes the context or relationships	Single or double-loop learning	Communication and collaborative engagement	Various techniques in support of communicative relationship with community	Dialogue	Be consulted
Shipton et al (2013)	Enhance competitive advantage and market performance	From	Service or product user	New insights from customers	Sustained competitive advantage and financial performance	Communication and collaborative engagement	Day-to-day experience	Increase participatory engagement; span boundaries	Allow engagement
Smith (2018)	Enhance competitive advantage and market performance	From	Service or product user	Information about customers	Improved service/product to attract customers	Feedback survey	Reflection, anticipation and problem solving	Collect information	Give information
Organizational learning documents									
Battor & Battour (2013)	Enhance competitive advantage and market performance	From	Service or product user	Detailed information about customer needs, market changes and competitor actions	Superior product or customer outcomes	Customer relationship management	Knowledge sharing	Maintain relationships with community; build learning capabilities	Not described

Citation	Motive to learn	With/from community	Community	What is learned	Learning outcomes	Structure for learning	Practice or process of learning	Role of organization	Role of community
Bess et al (2011)	Enhance capacity to create	From	Local geographic area	Relevant information for decision making	Increased organizational capacity	Participatory decision making	Relationship and dialogue	Provide a structure/ platform	Not described
Brockman et al (2017)	Enhance competitive advantage and market performance	From, with mediator	Service or product user	Information about customer needs and priorities	Improved market performance	Customer relationship management	Information sharing, interpretation and access	Collect and manage information; exploitation	Not described
Campbell (2003)	Enhance competitive advantage and market performance	From	Service or product user	Information about customer behaviour	Better service and more customers	Customer relationship management	Generation of knowledge from customers	Exploration and exploitation	Not described
Cegarra-Navarro & Dewhurst (2007)	Enhance competitive advantage and market performance	From	Service or product user	Information about problems faced by the organization and its customers	Enhanced customer capital	Unclear	Balanced exploration and exploitation	Exploration and exploitation	Use service
Chang (2019)	Improve new product development	From	Service or product user	Information about customer needs and product ideas	New, better products	New product development and testing	Knowledge management	Manage and apply knowledge during product development and testing	Participate
Curado et al (2019)	Enhance firm performance	With and from	Service or product user	Knowledge about, from and for customers; knowledge co-created with customers	Customized products, higher profitability, customer loyalty	Customer knowledge management	Exploration and exploitation	Integrate knowledge management with customer relationship management	Knowledge 'partner'

Citation	Motive to learn	With/from community	Community	What is learned	Learning outcomes	Structure for learning	Practice or process of learning	Role of organization	Role of community
Dahiyat & Al-Zu'bi (2012)	Enhance competitive advantage and market performance	With and from	Service or product user; knowledge partner	Information about customer situations, needs and wants	Competitive advantage in the market	Communication and collaborative engagement	Learning processes aim to explore, assimilate and exploit external knowledge	Maintain relationships with community; build learning capabilities	Participate
Desai (2018)	Refine internal routines or procedures and deepen organizational knowledge	With	Service or product user; municipality	Information about performance problems	Enhanced legitimacy of organization	Communication and collaborative engagement	Knowledge exchange, information search	Increase participatory engagement	Not described
Hafkesbrink & Schroll (2011)	Enhance competitive advantage and market performance	From	Service or product user	Information about consumer needs and reactions	User-informed service or product	Community of affinity	Reflective practice	Maintain relationships with community	Co-design, co-production; give feedback
Honig (2003)	Improve implementation of service or policy	From	Community agency	Information about partner goals, strategies and experiences	First- or second-order change, or knowledge in order not to change	Communication and collaborative engagement	Information search in the external environment and use	Exploration and exploitation	Not directly described
Jaziri (2019)	Enhance competitive advantage in the market	From	Service or product user	Information about the customer and customer experience	Product innovation	Customer experiential knowledge management	Absorption of customer experiential knowledge	Collect information about customer service experience	Experience the product through consumption
Li et al (2012)	Enhance competitive advantage and	From	Service or product user	Knowledge that is about, needed by, owned by or	Improved service/perfo rmance in the market	Unclear	Unclear	Not discussed	Not described

Citation	Motive to learn	With/from community	Community	What is learned	Learning outcomes	Structure for learning	Practice or process of learning	Role of organization	Role of community
	market performance			created by the customer					
Liu & Yuh-Yun Lin (2007)	Improve implementation of service or policy	From	Service or product user	Consumer data	Customer capital accumulation and healthy finances	Communication and engagement; information technology	Interaction	Exploration and exploitation	Drive innovation
Mitchell (2002)	Improve implementation of service or policy	From	Service or product user	Different ideas, perspectives and information not held by organization	Innovation, customer solutions, customize products	Communication and collaborative engagement; information technology	Generation of knowledge about and from customers	Collect information	Not described
Nonaka et al (2000)	Enhance competitive advantage and market performance	With	Service or product user	All kinds of tacit and explicit knowledge	Problem-solving and continuous creation of knowledge	A knowledge vision and knowledge management	Socialization, externalization, combination, internalization of knowledge	Build learning capabilities; promote learning structures and culture	Share tacit and explicit knowledges
Reay (2010)	Improve implementation of service or policy	From	Service or product user	Different perceptions of what counts as evidence	Informed service and changes in practice or policy	Communication and collaborative engagement	Learning occurs through research incorporating community views	Increase participatory engagement	Participate
Russell (2007)	Improve implementation of service or policy	With	Stakeholder/ interested party; community agency	Information about a threat	Conserve and empower	Government support	Problem response	Recognize community value	Be empowered to address problems

Citation	Motive to learn	With/from community	Community	What is learned	Learning outcomes	Structure for learning	Practice or process of learning	Role of organization	Role of community
Ryan (2018)	Not discussed	With and from	Community agency	Not described	Not described	Communication and collaborative engagement	External learning, decision-making, communication, operationalizing	Increase participatory engagement	Not described
Veronesi & Keasey (2015)	Unclear	With	Service or product user	Information about environment al and community needs	New partnerships, improved partner perceptions, strategic allocation of resources	Communication and collaborative engagement	Learning from mistakes and experimentation	Experiment	None
Wang & Xu (2018)	Improve implementation of service or policy	From	Service or product user	Explicit and tacit knowledge	Enhanced service innovation capability	Customer knowledge management	Open innovation	Collect and manage information	Co-design, co-production
Yanow (2004)	Learning is a by-product of daily experience	With and from	Any non-organization member	Information from daily experience	Not explicitly described	Communication and collaborative engagement	Knowledge translation	Value all knowledge	Do own thing
Yu & Jing (2008)	Enhance competitive advantage and market performance	With	Service or product user	Customer knowledge	Increased competitive advantage and new knowledge	Customer knowledge management	Knowledge sharing	Collect and manage information	Ask more
Ziyae et al (2019)	Enhance competitive advantage	From	Service or product user	Customer knowledge	Product innovation, increased competitive advantage	Customer knowledge management	Knowledge sharing	Collect and manage customer information	Not described

Citation	Motive to learn	With/from community	Community	What is learned	Learning outcomes	Structure for learning	Practice or process of learning	Role of organization	Role of community
Social learning documents									
Diduck et al (2012)	Solve problems	With and from	Public; stakeholder/ interested party	Information about tasks and values	Learning may provide an orientation toward greater social justice	Communication and collaborative engagement	Critical reflection, inquiry and transformation	Dialogue	Not directly described
Egunyu et al (2016)	Solve problems	From	Municipality; stakeholder/ interested party	Diverse knowledges, perspectives and values	Many outcomes, from new skills to changed values and assumptions	Policy-mandated collaboration	Deliberative processes	Increase participatory engagement	Provide knowledge Co-manage
Lundmark & Jonsson (2014)	Solve problems	With and from	Public; stakeholder/ interested party	Scientific and experiential knowledge	Improved implementati on and reduced conflict	Communication and collaborative engagement	Dialogue between groups offering different kinds of knowledge	Provide a structure/ platform	Participate
